# Training and burn care in rural India

**DOI:** 10.4103/0970-0358.70735

**Published:** 2010-09

**Authors:** Shobha Chamania

**Affiliations:** Department of Burn Surgery, Choithram hospital and Research Centre, Indore, India

**Keywords:** Kerosene, national programme, prevention, resource, training

## Abstract

Burn care is a huge challenge in India, having the highest female mortality globally due to flame burns. Burns can happen anywhere, but are more common in the rural region, affecting the poor. Most common cause is flame burns, the culprit being kerosene and flammable flowing garments worn by the women. The infrastructure of healthcare network is good but there is a severe resource crunch. In order to bring a positive change, there will have to be more trained personnel willing to work in the rural areas. Strategies for prevention and training of burn team are discussed along with suggestions on making the career package attractive and satisfying. This will positively translate into improved outcomes in the burns managed in the rural region and quick transfer to appropriate facility for those requiring specialised attention.

## INTRODUCTION

Burn care was primitive in India during 1970s–1980s. I was a trainee in general surgery in 1976–78 when I witnessed the treatment of burn patients by keeping at the end of the long ward, with no clear guidelines to treat them other than the initial resuscitation. Significant progress has been made from then to now: India has a few hundred surgeons and plastic surgeons committed/appointed to take care of burn patients in burn centre setting. Large majority of them are in urban regions. Physical infrastructure is developed with funding and trained personnel; but unfortunately, this does not ensure a standardised acute care for burns everywhere.

## RURAL EPIDEMIOLOGY

**Figure 1:**
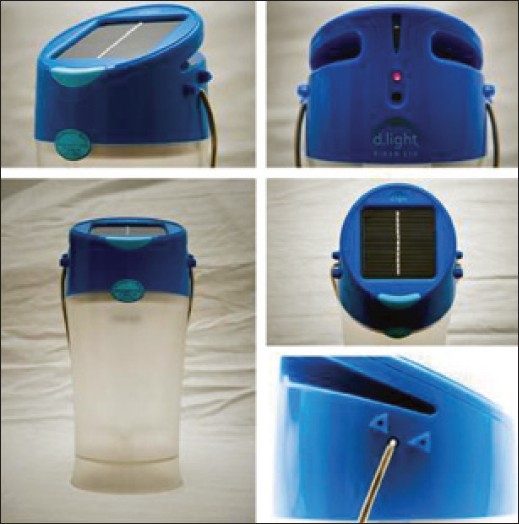
Solar lantern Kiran which is safer than kerosene lamp

Burns happen everywhere, but are somewhat more common in the rural areas. The main reason is the insufficient electric power supply leading to popular use of kerosene lamp for lighting which falls down frequently spilling kerosene and causing burns. Rural homes have very small or no windows; therefore, they need kerosene lamp during the day for illumination, especially in kitchen and toilets. The government of India has launched a solar lamp called Kiran[1] as an alternative, which is very safe [Figure 1]. It is a low-cost solar lighting solution, providing 8hours of light on full battery. It is four times brighter than kerosene lamp. It has been launched in Uttar Pradesh as a pilot and hopefully will be popularised nationally, saving many lives. There is also a Chinese solar lamp available in the market.

Kerosene stove continues to be another common cause of flame burns[2] as liquid petroleum gas supply to rural area is insufficient and biogas or wood as fuel has to be supplemented with quick lighting kerosene stove. The wick stove design was launched with safety standardisations but the females continue to suffer burns while cooking on stove. Unfortunately, there is a lot of ignorance about the method of extinguishing fire and the first aid to be given. This results in extensive burns with a significant proportion of full thickness burns.

Kerosene, a highly flammable liquid, is available in every household and unfortunately is used in most intentional burn injuries.

Rural women wear sari generally made of a polyester mix fabric which is highly flammable. Even a thin cotton sari is equally flammable. This is a flowing garment which catches fire from behind without the knowledge of the person wearing. This is followed by panic reaction when the person runs for help, adding further oxygen to fire. Panic reaction is a result of gross public ignorance about the right practice for dealing with a burning individual.

Children do get scalded due to floor level storing of hot liquids like water, milk and food.

Farmers get a lot of electrical burns during active agriculture phase when they try to get electricity from the mainline directly in order to pump water for the farm.

## HEATH CARE NETWORK IN INDIA

Rural healthcare has a network of primary health centres in the small towns (Tehsil or taluka) or villages, a district hospital in a district town and a referral medical college hospital in bigger cities of a state. Finally, there are state of the art centres providing specialised care in metros. These are the government organised networks nationally. Presently, they are focussed to provide maternity and child care, tuberculosis, malaria and AIDS control programmes. They are sparsely staffed in the rural and district regions, especially because the doctors do not find this as an attractive option. Better salaries and working conditions, along with the offer of reservation of candidates coming from rural region after 3 years of service for post graduation may invite attention of the doctors to the rural region.

Worldwide, there are 57 countries with a shortage of work force meant for critical healthcare. India is one among them. In its latest update, it is short of 74,000 accredited social health workers (ASHA) and 21,000 auxillary nurse midwives (ANMs).[3] My idea of citing these details of basic healthcare in India today is just to highlight that the network structure is in place but it is not adequately functional due to human and financial resource crunch, to even complete their basic health targets. Burn care will be an additional responsibility and it is a huge responsibility. Present infrastructure is not geared to accept fresh responsibilities.

District hospitals in a district headquarters do have surgeons and anaesthesiologists, but have an acute shortage of junior doctors, nurses and paramedical health workers. There is no facility available for taking care of burn patients. They are kept at the end of the general wards, under a mosquito net, with exposed wounds. There is no protocol of their management. I had an opportunity to visit a few district hospitals in Madhya Pradesh. They admit an average of 100 burn patients annually. Some are with fatal burns, >70% total body surface area (TBSA), who cannot afford to go to any other hospital. Then there are minor to moderate burns being cared for. Can one be satisfied that the minor to moderate burns are being attended to with no trained personnel or basic amenities?

Reviewing the state of the medical college hospitals (this is a general statement of the hospitals, excluding some of the excellent centres dedicated to acute burn care) has led to the following observations. They have a physical infrastructure of burn centre available but there is a huge lacuna in providing the basic standards of care for acutely burned, as they are not the priority on the list of any surgeon’s work plan. Patients can develop deformities while hospitalised and the care givers are not responsible or accountable for it. These are the training centres for future surgeons. If the trainee does not learn the correct system of burn care in the formative years, it is highly unlikely that he/she will be motivated to provide appropriate care or feel sorry for not providing, later in his career.

## STRATEGIES FOR TRAINING AND BURN CARE IN RURAL AREAS

Let us now focus on how we can positively help the unfortunate burn victim in the rural area. The healthcare giver, who is supposed to be giving health education to the community, should be trained for burn prevention and first aid. The message on extinguishing fire by stop, drop and roll, and then cool the burn by pouring tap water must take priority for educating rural masses. They must have educational material on safety with fire and first aid, targeting at the most important cause of burns in our country: flame burns by kerosene. These can include flip charts, posters, laptops with power point presentations in regional languages, sharing ghastly pictures of burned victims and their deformed outcomes and emphasising that preventing the burns is the best policy. The target audience should be children as well as adults, both men and women. Burn prevention messages should be heard/seen on radio and TV on a regular basis during prime time. These campaigns must teach people how accidents can be prevented by observing certain techniques and guidelines and they must also illustrate in detail what to do when accident occurs.[4] As Keswani said in 1986, “The challenge of burns in India lies not in the successful treatment of a 100% burn, but in the 100% prevention of all injuries”.[5]

Burn prevention should be a national programme, designed with sensitivity, vision and care towards advocacy of changing harmful and potentially dangerous cultural practices. Education must be combined with suggestions on some strategies of safe lifestyle for a common Indian. This will promote a lot of research in making their environment safe. Kiran solar lamp is an example of such research.

If we look at our neighbouring countries like Bangladesh, their rural burn epidemiology is also related to the floor level cooking outside home and the hot ash after cooking is left without cooling it. Children playing around this area get feet burns by stamping on hot ashes unknowingly. Mashreky *et al*.[6] from Center for Injury Prevention and Research, Dhaka, studied this problem affecting a large number of children and suggested a prevention plan which effectively separates rural children from the hot ashes by constructing a make shift barrier around the cooking area. They went ahead and offered help to house wives in the morning hours when the accidents were most common, by providing supervised child care. This has shown to reduce the incidence of burns significantly in the study region and now they are planning a national programme in liaison with the burn centre in Dhaka.

The state of rural burn care in Bangladesh and some African countries like Malawi, where international network for training, education and research in burns (Interburns) has had the opportunity to conduct Essential Burn Care Course (EBC), is very similar to India. These are the regions globally facing the biggest challenge of burns and fatality thereof, as reported by World Health Organization (WHO) global burden of injuries. These regions need collaboration from international burn fraternity, helping them to train, educate and encourage the right kind of research which will translate into motivated burn teams and improved outcomes.

## PREVENTION AS A COLLABORATIVE EFFORT: PUBLIC PRIVATE PARTNERSHIP

In 2006, Novartis’ Consumer Health and Sandoz divisions launched an initiative in India to address the neglected health needs of rural populations. The Arogya Parivar (healthy family) programme started with pilot sites in the states of Uttar Pradesh and Maharashtra. It combines healthcare education with access to affordable medicines through local pharmacies. Arogya Parivar health advisors speak to villagers about diseases and help them recognise symptoms. Periodic health camps bring in doctors to do examinations and make referrals to a treating doctor. A single health camp can attract from 200 to 2000 people. This is a social effort by Novartis India to reach out to the rural population. Arogya Parivar have reached out to cover a population of around 25 million villagers in seven states.[7]

They should become a collaborative partner for educating on burn prevention and first aid.

With good awareness on prevention and the correct first aid, in the long term, the incidence of major nonintentional burns can be reduced and the severity of burns will also be less. For those still injured, there should be clear guidelines for minor burns to be managed locally and others to be transported to a proper burn care facility. Transporting from remote villages and tribal areas can be a huge challenge and it may take them days to reach anywhere close to a facility, provided they are properly guided.

### Proposed guidelines

Very small superficial burns, not in critical areas, may be treated locally. Superficial partial thickness burns (are pink and moist, blanch on squeezing the non burned surrounding regions and are painful and sensate) which do not involve the face, hands, feet, perineum or circumferential area on the limbs (critical area burn) and there is no suspicion of inhalational injury then <10% surface burn in adult and <5% TBSA burn in children are very small burns hence can be treated in the local hospital. All burns in infants must be sent to a referral center for first consultation. There should be a training programme for these personnel to be oriented towards management of such injuries and they should be encouraged to get trained. This will prevent the post burn deformities, which form the main bulk of reconstructive work at present and affect the quality of outcome of a burned individual.

Burns of full thickness, involving a suspected inhalational component, present on critical areas and having other associated injuries or co-morbidities, which are more than 5% in an infant, and more than 10% in children, and more than 15% in an adult, should be referred to a burn care facility, for admission.

### Proposed training for burn team

This means that the primary healthcare giver should be conversant with assessment and minor burn wound management. They should also know what major burns are and how to deal with their life-saving emergencies and safely transfer them. This is the crying need of India and other countries like ours. During the transport the patient should have written instructions for the amount of fluid to be administered. In a conscious patient oral resuscitation should be encouraged. The medico legal formalities will have to be completed at the primary center before transfer and the patient must be shifted without unnecessary delay. Interburns has been focussing on this and has designed a special course called essential burn care course (EBC). Interburns trainer have a mix of representatives from burn centers of the resource rich and resource poor centers and includes representatives from all specialities. The trainees comprise of all team members and have trained 1500 people so far. There are many burn care courses globally; most are designed by the high resource countries for the resource rich infrastructures. These are irrelevant for low and middle income countries. The course designed by Interburns is for training the grass root workers for providing just the essential burn care. The focus is not critical care of major burns but salvaging minor to moderate burns in the facilities available locally. It is an interactive course and deals with all the aspects of burn care, from prevention to acute burn care and rehabilitation. The course is flexible and can be upgraded for physicians and down regulated for nurses and therapists with some specific changes. The course also emphasises that appropriate burn care is a matter of mindset; the training is to utilise the available resources to the fullest to achieve the objectives. If the government decides to train their health workers nationally, then the course is already designed and available for use. Interburns also runs an effective trainer course which can be used for developing the team of trainers countrywide.[8] District hospitals have voiced their needs for such trained personnel to start their campaign of caring for the burned.

On completion of this course, the participant should be able to:

- Understand and apply burn prevention strategies and run first-aid programmes
- Assess the burn patient’s needs
- Resuscitate and stabilise the burn patient
- Assess and manage minor burn wounds and their problems
- Organise basic burn care in a hospital and
- Understand and apply basic burn rehabilation

It is an established fact that an effective burn care facility has a multidisciplinary team to cater to their various needs. However, in the rural set-up, these trained individuals with the knowledge of essential burn care will be able to apply their skills to optimise the outcomes of minor burns. We need committed burn prevention at community level to increase public awareness about safety with fire and first aid. This can be performed by the nursing and medical students and interns during their community visits.

Surgeons need to be trained specifically for burn care after their post graduation. National Academy of Burns India (NABI) has a burn fellowship for any surgeon wanting to deal with burns in his practice. Recently, Interburns has also proposed a fellowship programme at Choithram Hospital and Research Centre, Indore, which has a 12-bed burn facility in a private trust. This will offer training for 6–8 weeeks. The learning objectives will be to get hands on training in acute burn care, from prevention to rehabilitation.

## CONCLUSION

It is a Herculean task to get the ball rolling in this huge country for improving healthcare in rural areas, where still the majority of Indian population resides, living their lives untouched by the progress made by the urban rich. Having recognised the strengths and weaknesses of our rural society, let us use this challenge of improving rural burn care as an opportunity for all those Indians involved in Burn care. It is our prerogative to educate and train masses and ensure that this education hopefully translates into desired outcome. We have to consider ourselves as privileged citizens in a country with 50% literacy rate, and therefore recognise that it is our duty to our society and nation to give back in the form of sharing our knowledge and experience. This will hopefully change the burn care scene in future, and the morbidities and mortalities will reduce. A good outcome in a patient is the best motivator for the burn team to continue to strive to improve further. This target will of course be achieved on the foundation pillars of effective and intensive prevention programme nationally and a patient friendly rehabilitation strategy.

The key points are as follows.

1. Burn incidence is very high in India and so is mortality and morbidity thereof. This affects everyone but rural population has to travel long distances in search of a burn care centre.
2. Most common cause of burn is flame burn due to kerosene. This is used in the lamps for illumination or stove for cooking. It is a domestic accident and females are the most common victim of burns.
3. There is a lot of ignorance about the first aid and prevention strategies, leading to higher incidence of major burns.
4. Presently, the rural health network is extremely strained. If trained personnel can be provided, the patients can be looked after well.
5. Training and education can be provided by Interburns through Essential burn care course.
6. Surgeons wishing to take care of burns should opt for a fellowship in burn care, conducted by NABI and Interburns.
7. Guidelines for managing minor burns in the rural set-up are suggested.
8. Burn prevention programme should be a national programme. This will ensure reduction in the incidence of burns.
